# Hemoglobin A_1c_ and Type 2 Diabetes Incidence Among Adolescents With Overweight and Obesity

**DOI:** 10.1001/jamanetworkopen.2023.51322

**Published:** 2024-01-17

**Authors:** Francis M. Hoe, Jeanne A. Darbinian, Louise C. Greenspan, Joan C. Lo

**Affiliations:** 1 Department of Pediatric Specialties, Kaiser Permanente Roseville Medical Center, Roseville, California; 2 The Permanente Medical Group, Oakland, California; 3 Division of Research, Kaiser Permanente Northern California, Oakland, California; 4 Department of Pediatrics, Kaiser Permanente San Francisco Medical Center, San Francisco, California

## Abstract

**Importance:**

With the increase in prediabetes among adolescents with overweight and obesity, identifying those at highest risk for type 2 diabetes (T2D) can support prevention strategies.

**Objective:**

To assess T2D risk by hemoglobin A_1c_ (HbA_1c_) levels among adolescents with overweight and obesity.

**Design, Setting, and Participants:**

This retrospective cohort study was conducted using data for January 1, 2010, to December 31, 2019, from a large California health care system. The study population comprised adolescents aged 10 to 17 years who had a body mass index (BMI; calculated as weight in kilograms divided by height in meters squared) at or above the 85th percentile, had HbA_1c_ measured during 2010 to 2018, and did not have preexisting diabetes. Data abstraction and analyses were conducted from January 1, 2020, to November 16, 2023.

**Exposures:**

Baseline HbA_1c_, with covariates including BMI category (overweight: 85th to <95th percentile; moderate obesity: 100% to <120% of 95th percentile; or severe obesity: ≥120% of 95th percentile), age, sex, race and ethnicity, and Neighborhood Deprivation Index score.

**Main Outcomes and Measures:**

The main outcome was incident T2D during follow-up through 2019, including cumulative incidence and multivariable hazard ratios (HRs) with 95% CIs using Cox proportional hazard regression analyses.

**Results:**

This study included 74 552 adolescents with a mean (SD) age of 13.4 (2.3) years. More than half (50.6%) were female; 26.9% of individuals had overweight, 42.3% had moderate obesity, and 30.8% had severe obesity. Individuals identified as Asian or Pacific Islander (17.6%), Black (11.1%), Hispanic (43.6%), White (21.6%), and other or unknown race or ethnicity (6.1%). During follow-up, 698 adolescents (0.9%) developed diabetes, and 626 (89.7%) had T2D; 72 individuals (10.3%) who had type 1, secondary, or other diabetes were censored. The overall T2D incidence was 2.1 (95% CI, 1.9-2.3) per 1000 person-years, with a 5-year cumulative incidence of 1.0% (95% CI, 0.9%-1.1%). Higher baseline HbA_1c_ (from <5.5% to 5.5%-5.6%, 5.7%-5.8%, 5.9%-6.0%, 6.1%-6.2%, and 6.3-6.4%) was associated with higher 5-year cumulative T2D incidence (from 0.3% [95% CI, 0.2%-0.4%] to 0.5% [0.4%-0.7%], 1.1% [0.8%-1.3%], 3.8% [3.2%-4.7%], 11.0% [8.9%-13.7%], and 28.5% [21.9%-36.5%], respectively). In addition, higher baseline HbA_1c_ was associated with greater T2D risk (reference [HbA_1c_ <5.5%]: HR, 1.7 [95% CI, 1.3-2.2], 2.8 [2.1-3.6], 9.3 [7.2-12.1], 23.3 [17.4-31.3], and 71.9 [51.1-101.1], respectively). Higher BMI category, older age, female sex, and Asian or Pacific Islander race (HR, 1.7 [95% CI, 1.3-2.2]), but not Black race or Hispanic ethnicity (compared with White race), were also independent indicators of T2D. In stratified analyses, incremental risk associated with higher HbA_1c_ was greater for Asian or Pacific Islander and White adolescents than for Black and Hispanic adolescents.

**Conclusions and Relevance:**

In this cohort study of adolescents with overweight and obesity, T2D risk increased substantially with baseline HbA_1c_ above 6.0%. Risk varied by BMI, age, sex, and race and ethnicity. These findings suggest that diabetes surveillance in adolescents should be tailored to optimize identification among high-risk subgroups.

## Key Points

**Question:**

What is the risk of future type 2 diabetes (T2D) among adolescents with overweight and obesity as indicated by hemoglobin A_1c_ (HbA_1c_) levels?

**Findings:**

In this cohort study of 74 552 adolescents aged 10 to 17 years with overweight or obesity, T2D incidence increased from 1 to 69 individuals per 1000 person-years as baseline HbA_1c_ increased from less than 5.5% to 6.3% to 6.4%, with the greatest increase beyond HbA_1c_ 6.0%. In multivariable analyses, T2D risk was 9-fold, 23-fold, and 72-fold higher for baseline HbA_1c_ levels of 5.9% to 6.0%, 6.1% to 6.2%, and 6.3% to 6.4%, respectively, compared with a baseline level below 5.5%.

**Meaning:**

These findings suggest that T2D surveillance in adolescents should be tailored based on HbA_1c_ level, among other risk factors.

## Teaser

This cohort study assesses the risk of type 2 diabetes by hemoglobin A1c (HbA_1c_) levels among adolescents with overweight and obesity.

## Introduction

Over the past 2 decades, prediabetes and type 2 diabetes (T2D) have increased among adolescents, parallel to the increase in childhood obesity.^1^ From 1999 to 2018, the prevalence of prediabetes in adolescents aged 12 to 19 years increased from 12% to 28%.^2^ From 2002 to 2015, the incidence of T2D in adolescents aged 10 to 19 years increased by 5% per year to 14 per 100 000 person-years.^3^ From 2001 to 2017, the prevalence of T2D increased by 95% to 67 per 100 000 individuals.^4^ Compared with adolescents with type 1 diabetes (T1D), adolescents with T2D have substantially greater cardiovascular risk burden (hypertension, obesity, or dyslipidemia) and microvascular complications (kidney disease, retinopathy, or neuropathy)^5,6,7^ and become young adults with much higher rates of cardiovascular disease and mortality.^8,9^ Thus, it is important to identify adolescents with the highest risk of developing T2D who may benefit from increased surveillance, targeted lifestyle intervention, and other treatment considerations before adulthood.

The American Diabetes Association (ADA) recommends diabetes screening for at-risk adolescents with overweight or obesity after pubertal onset or age 10 years, whichever occurs earlier, based on several criteria.^10,11^ These criteria include a body mass index (BMI; calculated as weight in kilograms divided by height in meters squared) at or above the 85th percentile for age and sex and at least 1 additional risk factor, such as a maternal history of diabetes during the child’s gestation, family history of T2D, race and ethnicity (American Indian or Alaska Native, Asian or Pacific Islander, Black, or Hispanic), or a sign or condition associated with insulin resistance (acanthosis nigricans, hypertension, dyslipidemia, polycystic ovarian syndrome, or small-for-gestational-age birth weight).^10,11^ These guidelines would currently target approximately one-quarter of US adolescents.^12^ Diabetes screening can be performed using fasting glucose, 2-hour glucose during an oral glucose tolerance test (OGTT), or hemoglobin A_1c_ (HbA_1c_).^10,11^ Of these screening measures, nonfasting HbA_1c_ assessment is more practical for adolescents because it is more convenient, time efficient, less variable, and more reproducible.^13^ In addition, use of HbA_1c_ results has been shown to increase diabetes screening among at-risk adolescents.^14^

A continuous association between HbA_1c_ and risk of T2D has been established in adults.^15,16^ Based on these observations, adults with an HbA_1c_ level of 5.7% to 6.4% are considered to have increased risk of developing T2D and are classified as having prediabetes.^11^ The ADA recommends these same HbA_1c_ thresholds to classify prediabetes in adolescents, although few population studies have validated the exact HbA_1c_ cut point of 5.7% in adolescents as indicative of future T2D.^10,11^ Data regarding T2D risk at higher HbA_1c_ levels are also limited and may be important for guiding screening frequency among adolescents with overweight and obesity, especially in racially and ethnically diverse populations. This study aimed (1) to determine T2D incidence by baseline HbA_1c_ levels in a diverse population of adolescents with overweight and obesity and (2) to identify clinically relevant HbA_1c_ thresholds associated with increased risk of T2D so that surveillance of high-risk populations can be optimized.

## Methods

### Design, Setting, and Study Population

This retrospective observational cohort study was conducted at Kaiser Permanente Northern California (KPNC) using electronic health record data (January 1, 2010, to December 31, 2019). The large KPNC integrated health care delivery system provides care to 4.4 million members in northern California, and approximately one-fifth of these individuals are aged younger than 20 years.^17^ The KPNC Institutional Review Board approved this study, and a waiver of informed consent was obtained [exemption category 4, with criteria §46.104(d)(4)(iii) met]. The study followed the Strengthening the Reporting of Observational Studies in Epidemiology (STROBE) reporting guideline.

The study cohort included KPNC members aged 10 to 17 years who had at least 1 HbA_1c_ measurement during 2010 to 2018 and a BMI at or above the 85th percentile for age and sex within the 12 months before or at the time of baseline HbA_1c_ measurement. Individuals were excluded if they had possible or confirmed preexisting diabetes, as evidenced by (1) a diagnosis of diabetes mellitus (*International Classification of Diseases, Ninth Revision, Clinical Modification* code 250.x or *International Classification of Diseases, Tenth Revision, Clinical Modification* codes E08.x to E13.x), (2) glycemic measurement in the diabetes range, or (3) receipt of glucose-lowering medication (eg, metformin, sulfonylurea, insulin, or other diabetes pharmacotherapy) before or at the time of baseline HbA_1c_ measurement based on pharmacy records. Individuals who were pregnant at the time of HbA_1c_ measurement were also excluded. A flowchart depicting identification and cohort assembly is shown in the eFigure in Supplement 1.

### Baseline Variables

Height and weight measurements from ambulatory visits were used to calculate BMI, with BMI percentiles for age and sex determined using the US Centers for Disease Control and Prevention growth chart reference data.^18^ The BMI categories for age and sex were defined as overweight (BMI 85th to <95th percentile) and obesity (BMI ≥95th percentile), subcategorized as moderate obesity (BMI 100% to <120% of 95th percentile) and severe obesity (BMI ≥120% of 95th percentile). Race and ethnicity was determined from patient- or family-reported data in health records and administrative databases and was classified as Asian or Pacific Islander, Black, Hispanic, non-Hispanic White (hereinafter White), or other race or ethnicity (including American Indian or Alaska Native, multiple races or ethnicities, or unknown race or ethnicity). These data were collected because T2D risk varies by race and ethnicity. Because neighborhood socioeconomic factors can influence T2D risk,^19^ we obtained Neighborhood Deprivation Index (NDI) scores, which ranged from −2.1 (lower estimated deprivation) to 4.4 (greater estimated deprivation) in our cohort. The NDI measure is derived using area of residence and US Census tract–level data relating to several socioeconomic factors (neighborhood wealth, income, education, occupation, and housing conditions).^20^

Baseline HbA_1c_ levels were reported from a single KPNC regional laboratory using the following analyzers: Modular P Tina-quant (2010), Integra 800 Tina-quant Gen. 2 (2011-2017), and Cobas c513 Tina-quant Gen. 3 (2017-2018; all from Roche Diagnostics), with all standardized to the National Glycohemoglobin Standardization Program (NGSP). During the period of use, the mean HbA_1c_ biases for each analyzer were −0.02, 0.07, and 0.01, respectively, compared with NGSP reference values between 5.4% and 6.4%.^21^ Using baseline HbA_1c_ data from this cohort, the mean (SD) HbA_1c_ levels for each analyzer were 5.35% (0.28%), 5.52% (0.26%), and 5.30% (0.26%), respectively. Other glycemic measures included fasting glucose, random glucose, and 2-hour glucose level during an OGTT. Diabetes range values were defined with HbA_1c_ (≥6.5%; 48 mmol/mol), fasting glucose (≥126 mg/dL; 7.0 mmol/L), random glucose (≥200 mg/dL; 11.1 mmol/L), or 2-hour glucose during an OGTT (≥200 mg/dL; 11.1 mmol/L) using ADA-recommended glycemic thresholds.^11^

### Outcome Ascertainment

Individuals with possible incident diabetes were initially identified from clinical diagnoses of diabetes by health care providers or any glycemic measure in the diabetes range occurring after baseline HbA_1c_ measurement. Medical record review was then conducted by a pediatric endocrinologist (F.M.H.) to identify individuals with incident diabetes, diagnosis date, and diabetes type. A random sample of 10% of individuals was also reviewed by a second pediatric endocrinologist (L.C.G.) to confirm diagnostic concordance. Individuals with incident diabetes were defined based on at least 1 glycemic measure in the diabetes range, using the aforementioned ADA glycemic thresholds (but not the ADA criteria of ≥2 glycemic measures in the diabetes range). To reduce false-positivity rates, fasting glucose levels of 126 to 199 mg/dL (7.0-11.0 mmol/L) were excluded if the following occurred: (1) the individual was documented to be not fasting or the test was performed after 12 pm, (2) repeat fasting glucose was less than 100 mg/dL (5.6 mmol/L), (3) 2-hour glucose during an OGTT was less than 140 mg/dL (7.8 mmol/L), or (4) HbA_1c_ was less than 5.9% within the following 2 weeks and diabetes was not subsequently confirmed. Random glucose at or above 200 mg/dL (11.1 mmol/L) was also excluded if measured in an inpatient, emergency department, or ambulatory procedure setting and diabetes was not subsequently confirmed, unless the encounter was related to diabetes.

Individuals were classified as having T1D if there was at least 1 positive diabetes autoantibody (eg, glutamic acid decarboxylase 65 [GAD65], insulinoma-associated protein 2, islet cell, or insulin autoantibodies) or based on physician-assigned diagnosis if diabetes autoantibodies were not measured (only in 7% of individuals with T1D). All other individuals were classified as having T2D except for those with maturity-onset diabetes of the young (MODY) or secondary diabetes. Secondary diabetes included steroid-induced hyperglycemia and cases related to chronic pancreatitis, asparaginase exposure, or hyperglycemia associated with major surgery. Individuals who developed gestational diabetes that resolved after pregnancy were not classified as having diabetes.

### Statistical Analysis

Baseline differences between subgroups were compared using analysis of variance for continuous variables and the χ^2^ test for categorical variables. Individuals were followed through 2019, with follow-up censored at membership disenrollment (gap >6 consecutive months), death, or development of diabetes. The Kaplan-Meier method was used to determine 5-year cumulative incidence of T2D by HbA_1c_ level. In addition, the T2D incidence rate was calculated per 1000 person-years with 95% CIs. Cox proportional hazard regression analyses were performed to examine the association of baseline HbA_1c_ and risk of T2D, accounting for sex, age, BMI category, race and ethnicity, and NDI quartile, reporting adjusted hazard ratios (HRs) and 95% CIs. Stratified analyses were also conducted by sex, BMI category, and race and ethnicity. In sensitivity analyses, we limited the multivariable analyses to the subset with at least 1 follow-up glycemic measure and additionally conducted analyses censoring at the last glycemic measure. All analyses were conducted using SAS, version 9.4 (SAS Institute Inc). A 2-sided *P* < .05 was used as the threshold for statistical significance. Data abstraction and analyses were conducted from January 1, 2020, to November 16, 2023.

## Results

We identified 103 800 adolescents (aged 10-17 years) with baseline HbA_1c_ measured in 2010 to 2018. Of these, 21 734 (20.9%) were excluded from this study due to BMI below the 85th percentile, 4345 (4.2%) due to age younger than 10 years at BMI measurement or no recent BMI measurement before HbA_1c_ assessment, and 3169 (3.1%) due to possible preexisting diabetes (3.0%) or gestational diabetes (0.1%) (eFigure in Supplement 1). The final analytic cohort included 74 552 adolescents aged 10 to 17 years with BMI at or above the 85th percentile and baseline HbA_1c_ of less than 6.5%. Table 1 presents demographic and baseline cohort characteristics by index HbA_1c_ level. Of the 74 552 included individuals, 49.4% were male and 50.6% were female; 64.6% were aged younger than 15 years and 73.1% had obesity. Individuals identified as Asian or Pacific Islander (17.6%), Black (11.1%), Hispanic (43.6%), White (21.6%), and other or unknown race or ethnicity (6.1%). Nearly a quarter of individuals (17 036 [22.9%]) had a baseline HbA_1c_ in the prediabetes range (5.7%-6.4%). The mean (SD) baseline HbA_1c_ varied by BMI category, increasing from 5.40% (0.27%) to 5.44% (0.27%) and 5.51% (0.29%) for adolescents with overweight, moderate obesity, and severe obesity, respectively (*P* < .001 for all pairwise comparisons). The mean (SD) baseline HbA_1c_ also varied by race and ethnicity, with values of 5.50% (0.28%) for Asian or Pacific Islander adolescents, 5.53% (0.32%) for Black adolescents, 5.45% (0.26%) for Hispanic adolescents, and 5.38% (0.26%) for White adolescents (*P* < .001 for all pairwise comparisons).

**Table 1.** Cohort Characteristics by Baseline HbA_1c_ Level^a^

Characteristic	Total cohort (N = 74 552)	Baseline HbA_1c_ level, %						*P* value^b^
		<5.5 (n = 36 949)	5.5-5.6 (n = 20 567)	5.7-5.8 (n = 11 707)	5.9-6.0 (n = 4138)	6.1-6.2 (n = 970)	6.3-6.4 (n = 221)	
Sex								
Male	36 859 (49.4)	17 650 (47.8)	10 498 (51.0)	6009 (51.3)	2123 (51.3)	486 (50.1)	93 (42.1)	<.001
Female	37 693 (50.6)	19 299 (52.2)	10 069 (49.0)	5698 (48.7)	2015 (48.7)	484 (49.9)	128 (57.9)	
Age, y								
Mean (SD)	13.4 (2.3)	13.6 (2.3)	13.3 (2.2)	13.2 (2.2)	13.1 (2.2)	13.2 (2.2)	13.6 (2.2)	<.001
10-11	19 892 (26.7)	9080 (24.6)	5779 (28.1)	3479 (29.7)	1228 (29.7)	276 (28.5)	50 (22.6)	<.001
12-14	28 274 (37.9)	13 400 (36.3)	8039 (39.1)	4673 (39.9)	1672 (40.4)	401 (41.3)	89 (40.3)	
15-17	26 386 (35.4)	14 469 (39.2)	6749 (32.8)	3555 (30.4)	1238 (29.9)	293 (30.2)	82 (37.1)	
BMI category^c^								
Overweight	20 037 (26.9)	11 287 (30.5)	5377 (26.1)	2494 (21.3)	733 (17.7)	122 (12.6)	24 (10.9)	<.001
Moderate obesity	31 547 (42.3)	16 110 (43.6)	8663 (42.1)	4803 (41.0)	1602 (38.7)	307 (31.6)	62 (28.0)	
Severe obesity	22 968 (30.8)	9552 (25.9)	6527 (31.7)	4410 (37.7)	1803 (43.6)	541 (55.8)	135 (61.1)	
Race and ethnicity								
Asian or Pacific Islander	13 131 (17.6)	5446 (14.7)	3847 (18.7)	2547 (21.8)	988 (23.9)	247 (25.5)	56 (25.3)	<.001
Black	8293 (11.1)	3172 (8.6)	2154 (10.5)	1734 (14.8)	891 (21.5)	277 (28.6)	65 (29.4)	
Hispanic	32 500 (43.6)	16 198 (43.8)	9325 (45.3)	5042 (43.1)	1544 (37.3)	327 (33.7)	64 (29.0)	
White	16 080 (21.6)	9836 (26.6)	4030 (19.6)	1666 (14.2)	460 (11.1)	69 (7.1)	19 (8.6)	
Other or unknown^d^	4548 (6.1)	2297 (6.2)	1211 (5.9)	718 (6.1)	255 (6.2)	50 (5.1)	17 (7.7)	
NDI quartile^e^								
1 (Least deprived)	18 559 (24.9)	9593 (26.0)	5025 (24.4)	2821 (24.1)	876 (21.2)	194 (20.0)	50 (22.6)	<.001
2	18 609 (25.0)	9457 (25.6)	5119 (24.9)	2785 (23.8)	968 (23.4)	231 (23.8)	49 (22.2)	
3	18 596 (24.9)	9036 (24.5)	5172 (25.2)	2981 (25.5)	1092 (26.4)	257 (26.5)	58 (26.2)	
4 (Most deprived)	18 569 (24.9)	8774 (23.7)	5184 (25.2)	3085 (26.3)	1183 (28.6)	280 (28.9)	63 (28.5)	
Unknown	219 (0.3)	89 (0.2)	67 (0.3)	35 (0.3)	19 (0.5)	8 (0.8)	1 (0.5)	

Abbreviations: BMI, body mass index (calculated as weight in kilograms divided by height in meters squared); HbA_1c_, hemoglobin A_1c_; NDI, Neighborhood Deprivation Index.

^a^ Unless stated otherwise, values are presented as No. (column %) of individuals.

^b^ The χ^2^ test or analysis of variance was used to examine overall differences for each variable across HbA_1c_ level.

^c^ Defined as overweight (BMI 85th to <95th percentile), moderate obesity (BMI 100% to <120% of 95th percentile), and severe obesity (BMI ≥120% of 95th percentile).

^d^ Includes American Indian or Alaska Native, multiple races or ethnicities, and unknown race or ethnicity.

^e^ Scores ranged from −2.1 to 4.4; higher scores represent greater estimated neighborhood deprivation. Scores were not available for 0.3% of children.

Individuals were followed from baseline HbA_1c_ measurement for a median duration of 3.5 (IQR, 1.8-5.9) years and a total of 300 711 person-years (eTable 1 in Supplement 1). The median (IQR) duration of follow-up was 2.8 (1.6-5.5), 3.8 (2.1-6.1), 4.2 (2.6-6.4), 4.3 (2.5-6.4), 4.0 (2.3-6.1), and 2.9 (1.4-5.2) years for baseline HbA_1c_ levels of less than 5.5%, 5.5% to 5.6%, 5.7% to 5.8%, 5.9% to 6.0%, 6.1% to 6.2%, and 6.3% to 6.4%, respectively. More than half of all individuals (55.7%) had 1 or more glycemic measurements performed during follow-up, including 216 (0.3%) with a 2-hour glucose during an OGTT. The median time from baseline HbA_1c_ to the last glycemic measure (HbA_1c_ in 63.0% of individuals) was 3.1 (IQR, 1.7-5.2) years. Of the 698 individuals (0.9%) who developed incident diabetes, 626 (89.7%) were classified as having T2D, with a median time to T2D diagnosis of 3.8 (IQR, 2.0-5.7) years. The number of individuals with T2D by baseline HbA_1c_, BMI category, sex, and race and ethnicity is shown in eTable 2 in Supplement 1. A total of 31.6% of individuals with T2D had 1 or more diabetes autoantibody titers measured (95.5% GAD65 antibody), and all had a negative result (eFigure in Supplement 1). Among the 95 individuals with T2D (15.2%) who received insulin therapy beyond the first 6 months postdiagnosis, 30 (4.3% of the 626 individuals with T2D) did not have diabetes autoantibody titers measured. All 30 individuals had obesity (25 had severe obesity): 13 started insulin at the time of T2D diagnosis, 17 started insulin after HbA_1c_ rose to 8% at a median time of 21 (range, 2-53) months postdiagnosis, and 4 discontinued insulin with subsequent HbA_1c_ less than 8% (eFigure in Supplement 1). An additional 72 individuals developed T1D, MODY, or secondary diabetes and were censored. Among the 57 individuals who developed T1D, 53 (93.0%) had diabetes autoantibody titers measured; all had at least 1 positive titer.

At 5 years, the cumulative incidence of T2D was 1.0% (95% CI, 0.9%-1.1%). The 5-year cumulative incidence of T2D when stratified by baseline HbA_1c_ level (<5.5%, 5.5% to 5.6%, 5.7% to 5.8%, and 5.9% to 6.0%) was 0.3%, (95% CI, 0.2%-0.4%), 0.5% (0.4%-0.7%), 1.1% (0.8%-1.3%), and 3.8% (3.2%-4.7%), respectively; it was considerably higher for higher HbA_1c_ levels (6.1%-6.2% and 6.3%-6.4%), at 11.0% (95% CI, 8.9%-13.7%) and 28.5% (21.9%-36.5%), respectively (Figure).

**Figure.**
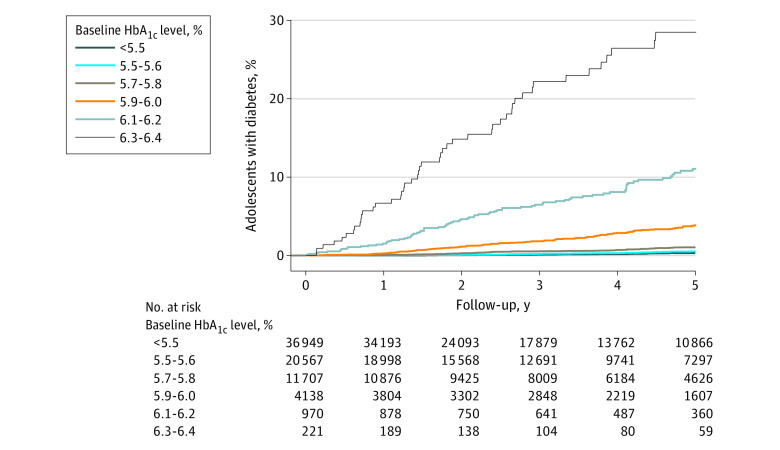
Cumulative Incidence of Type 2 Diabetes Over 5 Years of Follow-Up by Baseline Hemoglobin A_1c_ (HbA_1c_) Level

The overall incidence rate of T2D during follow-up was 2.1 (95% CI, 1.9-2.3) per 1000 person-years. As baseline HbA_1c_ increased (from <5.5% to 5.9%-6.0%, 6.1%-6.2%, and 6.3%-6.4%), T2D incidence increased exponentially from 0.8 (95% CI, 0.6-0.9) to 8.1 (6.8-9.5), 21.8 (17.5-26.8), and 68.9 (51.6-90.2) per 1000 person-years, respectively (Table 2). Incidence was higher in female adolescents (2.4 [95% CI, 2.1-2.6] vs 1.8 [1.6-2.0] per 1000 person-years) and increased with higher BMI category from 0.6 (95% CI, 0.5-0.8) to 1.3 (1.1-1.5) and 4.3 (3.9-4.7) per 1000 person-years among adolescents with overweight, moderate, and severe obesity, respectively. Adolescents with severe obesity had at least 2-fold higher incidence of T2D compared with adolescents with moderate obesity and even greater differences compared with adolescents with overweight, except at the highest baseline HbA_1c_ (6.3%-6.4%). Incidence per 1000 person-years also varied by race and ethnicity, with estimates of 3.0 (95% CI, 2.6-3.5) for Asian or Pacific Islander adolescents, 2.7 (2.2-3.4) for Black adolescents, 1.9 (1.7-2.2) for Hispanic adolescents, 1.3 (1.0-1.6) for White adolescents, and 1.7 (1.2-2.5) for adolescents of other or unknown race or ethnicity.

**Table 2.** Incidence Rate per 1000 Person-Years (95% CI) of Type 2 Diabetes by Baseline HbA_1c_ Level, Stratified by BMI Category, Race and Ethnicity, and Sex^a^

HbA_1c_, %	Incidence rate per 1000 person-years (95% CI)										
	Overall	BMI category^b^			Race and ethnicity					Sex	
		Overweight	Moderate obesity	Severe obesity	Asian or Pacific Islander	Black	Hispanic	White	Other^c^	Female	Male
Overall	2.1 (1.9-2.3)	0.6 (0.5-0.8)	1.3 (1.1-1.5)	4.3 (3.9-4.7)	3.0 (2.6-3.5)	2.7 (2.2-3.4)	1.9 (1.7-2.2)	1.3 (1.0-1.6)	1.7 (1.2-1.5)	2.4 (2.1-2.6)	1.8 (1.6-2.0)
<5.5	0.8 (0.6-0.9)	0.2 (0.1-0.4)	0.5 (0.3-0.7)	1.8 (1.4-2.3)	0.7 (0.4-1.1)	0.8 (0.4-1.5)	0.9 (0.7-1.2)	0.6 (0.4-0.9)	0.7 (0.2-1.6)	0.8 (0.6-1.1)	0.7 (0.5-0.9)
5.5-5.6	1.3 (1.0- 1.5)	0.4 (0.2-0.7)	0.9 (0.6-1.2)	2.6 (2.0-3.3)	1.8 (1.2-2.6)	1.1 (0.5-2.1)	1.3 (1.0-1.7)	0.9 (0.5-1.4)	0.9 (0.2-2.3)	1.5 (1.1-1.9)	1.1 (0.8-1.4)
5.7-5.8	2.3 (1.9-2.8)	0.6 (0.3-1.3)	1.8 (1.3-2.5)	3.8 (3.0-4.7)	3.3 (2.3-4.5)	2.0 (1.1-3.2)	2.0 (1.5-2.7)	2.4 (1.4-3.8)	1.4 (0.4-3.5)	2.8 (2.2-3.5)	1.8 (1.4-2.4)
5.9-6.0	8.1 (6.8-9.5)	3.3 (1.7-5.9)	5.9 (4.3-8.0)	12.2 (9.8-14.9)	9.1 (6.5-12.3)	7.9 (5.4-11.3)	7.6 (5.7-10.0)	9.2 (5.5-14.3)	5.1 (1.6-11.8)	9.4 (7.5-11.7)	6.8 (5.3-8.7)
6.1-6.2	21.8 (17.5-26.8)	12.1 (4.4-26.4)	10.6 (5.9-17.4)	31.4 (24.4-39.8)	19.8 (12.4-30.0)	18.7 (11.7-28.4)	25.5 (17.5-35.8)	24.6 (9.9-50.7)	23.1 (7.5-53.9)	24.9 (18.5-32.8)	18.8 (13.4-25.8)
6.3-6.4	68.9 (51.6-90.2)	86.9 (35.0-179.1)	56.7 (30.2-97.0)	71.9 (49.5-101.0)	121.1 (74.9-185.1)	23.2 (8.5-50.5)	66.9 (38.2-108.6)	94.9 (30.8-221.5)	111.3 (36.1-259.8)	68.8 (46.4-98.3)	69.1 (43.8-103.6)

Abbreviations: BMI, body mass index (calculated as weight in kilograms divided by height in meters squared); HbA_1c_, hemoglobin A_1c_.

^a^ Table 1 provides the number of individuals and eTables 1 and 2 in Supplement 1 provide the duration of follow-up and number of individuals in each category.

^b^ Defined as overweight (BMI 85th to <95th percentile), moderate obesity (BMI 100% to <120% of 95th percentile), and severe obesity (BMI ≥120% of 95th percentile).

^c^ Includes American Indian or Alaska Native, multiple races or ethnicities, and unknown race or ethnicity.

In multivariable analyses, adjusting for age, sex, race and ethnicity, BMI category, and NDI quartile, higher baseline HbA_1c_ was associated with an exponential increase in T2D risk (Table 3), with 72-fold increased risk (HR, 71.9 [95% CI, 51.1-101.1]) for HbA_1c_ of 6.3% to 6.4% compared with HbA_1c_ of less than 5.5%. Moderate (HR, 2.0 [95% CI, 1.4-2.7]) and severe (5.2 [3.9-7.1]) obesity (vs overweight), female sex (1.5 [1.3-1.8]), older age (1.7 [1.4-2.1] for ages 15-17 vs 10-11 years), and Asian or Pacific Islander race (1.7 [1.3-2.2] compared with White), but not Black race (0.8 [0.6-1.1]) or Hispanic ethnicity (1.1 [0.8-1.4]), were independently associated with T2D risk. Findings were similar in sensitivity analyses that excluded adolescents with no follow-up glycemic measure and additionally censored follow-up time at the last glycemic measure (Table 3). In analyses stratified by race and ethnicity (Table 4), T2D risk increased with increasing HbA_1c_ levels for all groups, but the magnitude of increase at higher HbA_1c_ levels tended to be less for Black and Hispanic adolescents compared with Asian or Pacific Islander and White adolescents.

**Table 3.** Multivariable Association of HbA_1c_ Levels and Risk of Type 2 Diabetes^a^

Characteristic	HR (95% CI)		
	Entire cohort (N = 74 552)	Subset with glycemic follow-up measure (n = 41 541)	Subset censored at last glycemic measure (n = 41 541)
**Unadjusted**			
HbA_1c_, %			
<5.5	1 [Reference]	1 [Reference]	1 [Reference]
5.5-5.6	1.7 (1.3-2.3)	1.7 (1.3-2.2)	1.8 (1.4-2.3)
5.7-5.8	3.2 (2.4-4.1)	2.9 (2.2-3.7)	3.2 (2.4-4.1)
5.9-6.0	11.2 (8.7-14.4)	9.8 (7.6-12.6)	10.6 (8.2-13.7)
6.1-6.2	30.9 (23.2-41.0)	25.3 (19.0-33.6)	26.7 (20.1-35.5)
6.3-6.4	103.4 (74.1-144.1)	81.4 (58.3-113.5)	90.5 (64.8-126.4)
**Adjusted**			
HbA_1c_, %			
<5.5	1 [Reference]	1 [Reference]	1 [Reference]
5.5-5.6	1.7 (1.3-2.2)	1.6 (1.2-2.1)	1.7 (1.3-2.3)
5.7-5.8	2.8 (2.1-3.6)	2.6 (2.0-3.4)	2.9 (2.2-3.8)
5.9-6.0	9.3 (7.2-12.1)	8.4 (6.5-10.8)	8.9 (6.9-11.5)
6.1-6.2	23.3 (17.4-31.3)	20.8 (15.5-27.9)	22.3 (16.6-29.9)
6.3-6.4	71.9 (51.1-101.1)	62.3 (44.3-87.7)	68.9 (48.9-97.0)
Sex			
Female	1.5 (1.3-1.8)	1.4 (1.2-1.6)	1.2 (1.0-1.4)
Male	1 [Reference]	1 [Reference]	1 [Reference]
Age, y			
10-11	1 [Reference]	1 [Reference]	1 [Reference]
12-14	1.0 (0.8-1.2)	1.0 (0.8-1.2)	1.0 (0.8-1.3)
15-17	1.7 (1.4-2.1)	1.9 (1.5-2.3)	1.9 (1.5-2.3)
BMI category^b^			
Overweight	1 [Reference]	1 [Reference]	1 [Reference]
Moderate obesity	2.0 (1.4-2.7)	1.9 (1.4-2.7)	1.9 (1.4-2.6)
Severe obesity	5.2 (3.9-7.1)	5.0 (3.7-6.7)	4.6 (3.4-6.2)
Race and ethnicity			
Asian or Pacific Islander	1.7 (1.3-2.2)	1.7 (1.3-2.3)	1.7 (1.3-2.3)
Black	0.8 (0.6-1.1)	0.8 (0.6-1.0)	0.8 (0.6-1.0)
Hispanic	1.1 (0.8-1.4)	1.0 (0.8-1.4)	1.0 (0.8-1.3)
White	1 [Reference]	1 [Reference]	1 [Reference]
Other or unkown^c^	1.0 (0.6-1.5)	1.0 (0.7-1.6)	1.1 (0.7-1.7)
NDI quartile^d^			
1 (Least deprived)	1 [Reference]	1 [Reference]	1 [Reference]
2	1.0 (0.8-1.2)	1.0 (0.8-1.2)	1.0 (0.8-1.3)
3	1.2 (1.0-1.5)	1.2 (1.0-1.6)	1.3 (1.0-1.6)
4 (Most deprived)	1.2 (1.0-1.5)	1.3 (1.0-1.6)	1.4 (1.1-1.7)
Unknown	1.0 (0.3-4.2)	1.1 (0.3-4.3)	1.2 (0.3-4.8)

Abbreviations: BMI, body mass index (calculated as weight in kilograms divided by height in meters squared); HbA_1c_, hemoglobin A_1c_; HR, hazard ratio; NDI, Neighborhood Deprivation Index.

^a^ Sensitivity analyses were conducted, restricting the cohort to those with a follow-up glycemic measure and to those with a follow-up glycemic measure, censored at the last glycemic measure.

^b^ Defined as overweight (BMI 85th to <95th percentile), moderate obesity (BMI 100% to <120% of 95th percentile), and severe obesity (BMI ≥120% of 95th percentile).

^c^ Includes American Indian or Alaska Native race, multiple races or ethnicities, and unknown race or ethnicity.

^d^ Scores ranged from −2.1 to 4.4; higher scores represent greater estimated neighborhood deprivation. Scores were not available for 0.3% of children.

**Table 4.** Multivariable Association of HbA_1c_ Levels and Risk of Type 2 Diabetes by Race and Ethnicity

Characteristic	HR by race and ethnicity (95% CI)				
	Asian or Pacific Islander	Black	Hispanic	White	Other or unkown^a^
HbA_1c_, %					
<5.5	1 [Reference]	1 [Reference]	1 [Reference]	1 [Reference]	1 [Reference]
5.5-5.6	2.8 (1.5-5.2)	1.3 (0.6-3.2)	1.4 (1.0-2.1)	1.7 (0.9-3.2)	1.4 (0.4-5.1)
5.7-5.8	4.7 (2.5-8.7)	2.0 (0.9-4.5)	2.0 (1.3-3.0)	4.6 (2.4-8.7)	2.1 (0.6-7.8)
5.9-6.0	12.4 (6.7-22.8)	7.9 (3.8-16.3)	7.4 (5.0-11.0)	15.1 (8.0-28.5)	7.2 (2.1-25.4)
6.1-6.2	25.6 (13.0-50.4)	16.1 (7.5-34.5)	22.0 (14.1-34.2)	42.0 (17.5-100.4)	35.7 (9.6-132.4)
6.3-6.4	155.2 (77.7-310.3)	21.5 (7.7-60.2)	49.2 (28.0-86.7)	146.0 (52.5-406.5)	143.9 (37.1-558.6)
Sex					
Female	2.0 (1.5-2.7)	1.2 (0.8-1.8)	1.4 (1.1-1.8)	1.3 (0.9-2.1)	2.7 (1.2-6.4)
Male	1 [Reference]	1 [Reference]	1 [Reference]	1 [Reference]	1 [Reference]
Age, y					
10-11	1 [Reference]	1 [Reference]	1 [Reference]	1 [Reference]	1 [Reference]
12-14	0.8 (0.6-1.2)	0.8 (0.5-1.4)	1.2 (0.9-1.7)	0.8 (0.4-1.6)	1.8 (0.6-5.5)
15-17	1.3 (0.9-1.8)	1.2 (0.7-1.9)	2.3 (1.6-3.2)	1.7 (0.9-3.1)	2.8 (1.0-8.2)
BMI category^b^					
Overweight	1 [Reference]	1 [Reference]	1 [Reference]	1 [Reference]	1 [Reference]
Moderate obesity	2.0 (1.2-3.4)	2.2 (0.6-7.6)	2.2 (1.2-3.9)	1.3 (0.6-2.8)	4.1 (0.9-19.6)
Severe obesity	4.0 (2.5-6.6)	7.8 (2.4-25.0)	6.3 (3.6-11.0)	4.1 (2.0-8.3)	7.0 (1.6-30.9)
NDI quartile^c^					
1 (Least deprived)	1 [Reference]	1 [Reference]	1 [Reference]	1 [Reference]	1 [Reference]
2	0.9 (0.6-1.4)	0.7 (0.4-1.4)	0.7 (0.5-1.2)	1.9 (1.1-3.2)	0.6 (0.2-1.7)
3	1.5 (1.0-2.3)	0.8 (0.5-1.5)	1.0 (0.7-1.5)	1.8 (1.0-3.2)	0.9 (0.3-2.3)
4 (Most deprived)	1.5 (1.0-2.4)	0.8 (0.4-1.4)	1.1 (0.8-1.6)	1.3 (0.6-2.9)	0.5 (0.1-1.6)

Abbreviations: BMI, body mass index (calculated as weight in kilograms divided by height in meters squared); HbA_1c_, hemoglobin A_1c_; HR, hazard ratio; NDI, Neighborhood Deprivation Index.

^a^ Includes American Indian or Alaska Native race, multiple races or ethnicities, and unknown race or ethnicity.

^b^ Defined as overweight (BMI 85th to <95th percentile), moderate obesity (BMI 100% to <120% of 95th percentile), and severe obesity (BMI ≥120% of 95th percentile).

^c^ Scores ranged from −2.1 to 4.4; higher scores represent greater estimated neighborhood deprivation. Scores were not available for 0.3% of children.

## Discussion

In a cohort of 74 552 adolescents followed for more than 300 000 person-years, 626 individuals with incident T2D were identified, representing one of the largest single-center studies to date examining incidence of T2D among adolescents with overweight and obesity. Our cohort size, which included 17 036 adolescents with baseline HbA_1c_ in the prediabetes range (5.7%-6.4%), allowed for detailed examination of T2D risk by incremental levels of baseline HbA_1c_, which was not possible in prior studies with smaller cohort size.^22,23,24^ We observed that baseline HbA_1c_ level was a strong indicator of incident T2D. As baseline HbA_1c_ increased (from <5.5% to 5.9%-6.0%, 6.1%-6.2%, and 6.3%-6.4%), the 5-year cumulative incidence of T2D increased exponentially from 0.3% to 3.8%, 11.0%, and 28.5%, respectively. Accounting for demographic characteristics and baseline BMI levels, the risk of T2D was 9-fold, 23-fold, and 72-fold higher, respectively.

The overall incidence of T2D among adolescents with overweight and obesity was low (2.1 per 1000 person-years) and was generally low even among adolescents with baseline HbA_1c_ in the lower prediabetes range (HbA_1c_, 5.7%-6.0%), in which the T2D incidence was 3.8 per 1000 person-years. However, larger incremental differences in risk were apparent for baseline HbA_1c_ above 6.0% and support findings from a previous study that identified 368 predominantly Hispanic adolescents with overweight or obesity and baseline HbA_1c_ of 5.7% to 6.4%, in which the incidence of subsequent diabetes (HbA_1c_ ≥6.5%) increased from 25 to 50 per 1000 person-years for baseline HbA_1c_ of 5.7% to 5.9% and 6.0% to 6.4%, respectively.^22^ Our study also identified other independent risk factors for T2D, including older age, female sex, higher BMI, and Asian or Pacific Islander race, consistent with previous studies of T2D in adolescents.^3,4,23,25,26,27,28,29,30^ Although Black and Hispanic adolescents in this study had higher mean baseline HbA_1c_, similar to trends in previous studies,^31,32,33^ they did not have a higher risk of T2D after adjusting for baseline HbA_1c_ level.

The US Preventive Services Task Force recently stated that “current evidence is insufficient to assess the balance of benefits and harms of screening for T2D in children and adolescents” and “more studies are needed… [to identify] factors associated with risk of progression to diabetes.”^34^ The current study adds compelling data that adolescents with HbA_1c_ levels of 6.1% to 6.4% comprise a much higher-risk subset that would benefit from regular diabetes screening. Adolescents with HbA_1c_ levels of 5.7% to 5.8%, who comprised two-thirds (68.7%) of those with HbA_1c_ in the prediabetes range in the current study, had a relatively low incidence of T2D (2.3 per 1000 person-years) and could be considered for follow-up diabetes screening less frequently than once per year, as currently recommended by the ADA for individuals with prediabetes.^35^

### Limitations and Strengths

This study had several limitations. First, we cannot exclude the possibility of selection bias regarding who had baseline HbA_1c_ testing. Hemoglobin A_1c_ can also be affected by health conditions (eg, hemoglobinopathies, anemia, and glucose-6-phosphate dehydrogenase deficiency), which were not examined in this study.^11,13^ Second, we required only 1 glycemic measure in the diabetic range to define incident diabetes, rather than 2 glycemic measures, similar to other large epidemiologic studies.^12,36^ Third, 44.3% of individuals did not have a follow-up glycemic measure performed (required for T2D diagnosis), but sensitivity analyses restricted to those with at least 1 follow-up glycemic measure showed similar findings. Nonetheless, we cannot exclude potential follow-up bias and T2D underdiagnosis. Fourth, although many individuals with T2D did not have diabetes autoantibodies measured, only a very small subset (4.2%) persistently required insulin therapy, where we cannot entirely exclude the possibility of T1D. Finally, other factors that can influence risk of T2D, such as change in weight or BMI, cardiometabolic conditions, and pubertal timing (incompletely captured during routine care), were not examined.

A major strength of our study is the inclusion of a large and diverse cohort receiving care in the same integrated health care setting where laboratory and pharmacy data, as well as clinical diagnoses, could be tracked in electronic health records. Our findings are notable in that 15.2% of individuals who developed T2D required insulin beyond 6 months of diagnosis. Previous studies have also shown that about half of adolescents with T2D eventually require insulin and 10% present with diabetic ketoacidosis (with even higher rates of both among individuals with new-onset T2D during the COVID-19 pandemic), reinforcing the notion that adolescents with T2D have a higher degree of insulin deficiency compared with adults.^37,38,39,40^

## Conclusions

Among the ethnically diverse adolescents with overweight and obesity in this cohort study, the incidence of T2D was relatively low but increased with increasing baseline HbA_1c_, particularly at HbA_1c_ levels above 6.0%. Although HbA_1c_ was a strong indicator of T2D, risk was also associated with obesity severity, age, female sex, and Asian or Pacific Islander race. Hence, T2D surveillance in adolescents should be primarily based on HbA_1c_ but should also consider these other risk factors when optimizing prevention strategies for those at highest risk. Research is needed to determine which interventions (eg, lifestyle intervention, pharmacotherapy, or other treatment) are most effective in preventing progression to T2D among those at highest risk.

## Notes

Supplement 1. **eFigure.** Inclusion and Exclusion Criteria for Cohort **eTable 1.** Duration of Follow-Up (Person-Years) by Baseline Hemoglobin A_1c_ (HbA_1c_) Level, Stratified by Body Mass Index (BMI) Category, Race and Ethnicity, and Sex **eTable 2.** Incident Cases of Type 2 Diabetes by Baseline Hemoglobin A_1c_ (HbA_1c_) Level, Stratified by Body Mass Index (BMI) Category, Race and Ethnicity, and Sex

Supplement 2.
Data Sharing Statement
