# Supplementary material for: Hemoglobin A1c and Type 2 Diabetes Incidence Among Adolescents With Overweight and Obesity
Source: JAMA Netw Open. 2024 Jan 17;7(1):e2351322. doi: 10.1001/jamanetworkopen.2023.51322 (PMC10794942; doi:10.1001/jamanetworkopen.2023.51322)
Supplement: Supplement 1. — eFigure. Inclusion and Exclusion Criteria for Cohort eTable 1. Duration of Follow-Up (Person-Years) by Baseline Hemoglobin A1c (HbA1c) Level, Stratified by Body Mass Index (BMI) Category, Race and Ethnicity, and Sex eTable 2. Incident Cases of Type 2 Diabetes by Baseline Hemoglobin A1c (HbA1c) Level, Stratified by Body Mass Index (BMI) Category, Race and Ethnicity, and Sex [file jamanetwopen-e2351322-s001.pdf]

Supplementary Online Content

Hoe FM, Darbinian JA, Greenspan LC, Lo JC. Hemoglobin A_{1c} and type 2 diabetes incidence among adolescents with overweight and obesity. *JAMA Netw Open*. 2024;7(1):e2351322. doi:10.1001/jamanetworkopen.2023.51322

eFigure. Inclusion and Exclusion Criteria for Cohort

eTable 1. Duration of Follow-Up (Person-Years) by Baseline Hemoglobin A_{1c} (HbA_{1c}) Level, Stratified by Body Mass Index (BMI) Category, Race and Ethnicity, and Sex

eTable 2. Incident Cases of Type 2 Diabetes by Baseline Hemoglobin A_{1c} (HbA_{1c}) Level, Stratified by Body Mass Index (BMI) Category, Race and Ethnicity, and Sex

This supplementary material has been provided by the authors to give readers additional information about their work.

© 2024 Hoe FM et al. *JAMA Network Open.*

eFigure. Inclusion and Exclusion Criteria for Cohort

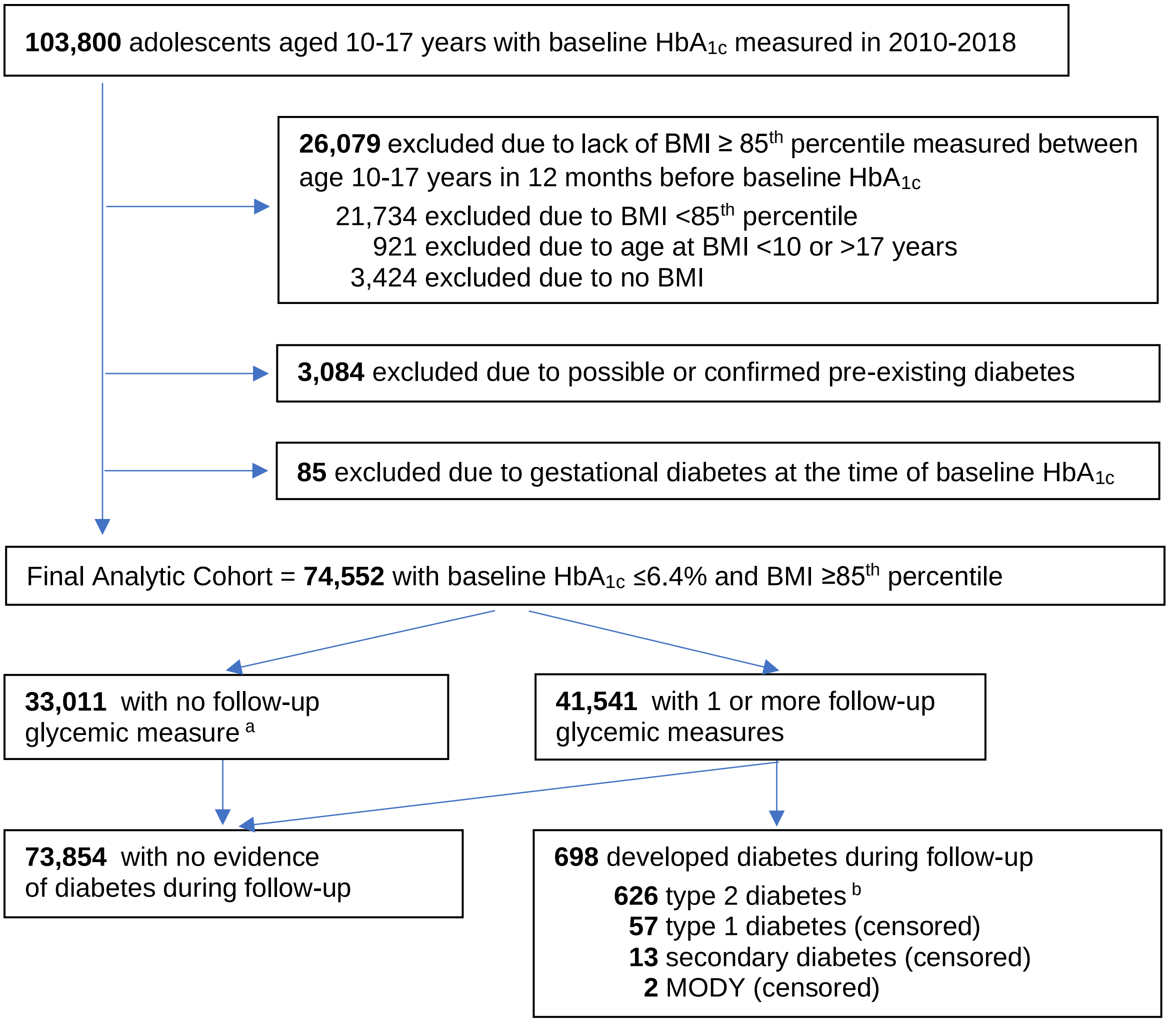

[a] Subjects with no follow-up glycemic measure, compared to those with ≥1 follow-up glycemic measure, were more likely to have baseline HbA_{1c} measured during the last 3 years of cohort entry, 2016-2018 (54.2% vs 29.2%, $p<0.001$).

[b] Among the 626 adolescents who developed type 2 diabetes (T2D):

- 198 (31.6%) had negative diabetes autoantibodies (Ab) measured (95.5% GAD65 Ab)
- 95 (15.2%) on insulin beyond 6 months after T2D diagnosis
  - 65 (10.4% of 626 adolescents who developed T2D) had negative diabetes Ab measured
    - 39 started insulin at diagnosis
    - 26 started insulin after HbA_{1c} >8% (median 23 months after diagnosis, range 7-68)
    - 4 discontinued insulin with subsequent HbA_{1c} <8%
  - 30 (4.8% of 626 adolescents who developed T2D) had no diabetes Ab measured.
    - All had obesity (25 had severe obesity)
    - 13 started insulin at diagnosis
    - 17 started insulin after HbA_{1c} >8% (median 21 months after diagnosis, range 2-53)
    - 4 discontinued insulin with subsequent HbA_{1c} <8%

© 2023 Hoe FM et al. *JAMA Network Open.*

eTable 1. Duration of Follow-Up (Person-Years) by Baseline Hemoglobin A_{1c} (HbA_{1c}) Level, Stratified by Body Mass Index (BMI) Category, Race and Ethnicity, and Sex

HbA_{1c} (%)	Overall	BMI Category			Race and Ethnicity					Sex	
		Overweight	Moderate Obesity	Severe Obesity	Asian or Pacific Islander	Black	Hispanic	White	Other or unknown[a]	Female	Male
Overall	300,711	78,555	127,050	95,106	55,081	34,193	129,783	65,545	16,109	151,549	149,162
<5.5	139,408	40,931	61,029	37,448	21,038	12,361	60,515	38,056	7,439	72,786	66,622
5.5 to 5.6	86,187	22,568	36,197	27,422	16,603	8,810	38,631	17,611	4,531	42,073	44,114
5.7 to 5.8	52,002	11,153	21,054	19,795	11,533	7,688	22,425	7,468	2,888	25,335	26,667
5.9 to 6.0	18,265	3,327	7,121	7,817	4,624	3,901	6,677	2,073	990	8,908	9,357
6.1 to 6.2	4,080	495	1,420	2,165	1,109	1,174	1,296	284	216	2,011	2,069
6.3 to 6.4	769	81	229	459	174	259	239	53	45	436	333

[a] Includes Native American/Alaska Native, multiracial, and unknown race and ethnicity

eTable 2. Incident Cases of Type 2 Diabetes by Baseline Hemoglobin A_{1c} (HbA_{1c}) Level, Stratified by Body Mass Index (BMI) Category, Race and Ethnicity, and Sex

HbA_{1c} (%)	Overall	BMI Category			Race and Ethnicity					Sex	
		Overweight	Moderate Obesity	Severe Obesity	Asian or Pacific Islander	Black	Hispanic	White	Other or unknown[a]	Female	Male
Overall	626	49	168	409	167	94	251	86	28	357	269
<5.5	106	10	29	67	14	10	55	22	5	60	46
5.5 to 5.6	110	8	31	71	30	10	51	15	4	62	48
5.7 to 5.8	120	7	38	75	38	15	45	18	4	71	49
5.9 to 6.0	148	11	42	95	42	31	51	19	5	84	64
6.1 to 6.2	89	6	15	68	22	22	33	7	5	50	39
6.3 to 6.4	53	7	13	33	21	6	16	5	5	30	23

[a] Includes Native American/Alaska Native, multiracial, and unknown race and ethnicity

© 2023 Hoe FM et al. *JAMA Network Open.*
