# Supplementary material for: Hemoglobin A1c and Type 2 Diabetes Incidence Among Adolescents With Overweight and Obesity
Source: JAMA Netw Open. 2024 Jan 17;7(1):e2351322. doi: 10.1001/jamanetworkopen.2023.51322 (PMC10794942; doi:10.1001/jamanetworkopen.2023.51322)
Supplement: Supplement 2. — Data Sharing Statement [file jamanetwopen-e2351322-s002.pdf]

Data Sharing Statement

Hoe. Hemoglobin A_{1c} and Type 2 Diabetes Incidence Among Adolescents With Overweight and Obesity. *JAMA Netw Open*. Published January 17, 2024.
doi:10.1001/jamanetworkopen.2023.51322

Data
Data available: No

Additional Information
Explanation for why data not available: Our research protocol does not allow for public dissemination of the datasets used in this study.
